# Service, Prof. Wm. H. Byford, M. D., Attending Physician

**Published:** 1861-11

**Authors:** 


**Service, Prof. Wm. H. BYFORD, M. D., Attending Physician.**

Reported for the EXAMINER.

*Ozœna.*—GENTLEMEN :—Inflammation affects the nasal passage in different degrees of intensities,—from the slight catarrh, to the more destructive forms of ozœna, in which parts of the mucous membrane, the periosteum, and not unfrequently, even the bones are destroyed. Exfoliation of the bones often takes place, to such an extent as to let down the bridge of the nose, and, by removing the septum, lay both cavities into one great cavern; while the breath becomes intolerably fœtid, and acrid discharges keep the lips and alæ nasi, chapped, excoriated and ulcerated. These severe forms of inflammation of the contents of the nasal passages, are apt to be connected with some specific taint in the system. Syphilis, in the very young infant, shows itself, often a few days after birth, in the form of ozœna or "snuffles," as it is called in these young patients. As you have seen, doubtless, in the wards of the Marine Hospital, ozœna is a troublesome symptom in some of the syphilitic patients, so generally exhibited to you there. In scrofulous persons, too, inflammation of the nasal cavities is apt to become an obstinate affection; and it is not rare to find patients, troubled with this form of disease in middle, and even advanced life, who date the commencement of their disease from early childhood. Scrofula is a vague term, and we are apt to attribute to it too many forms of disease, because we know of no more plausible and definite causation.

This boy, apparently in the enjoyment of good health and fair vigor of constitution, came to me, three days ago, complaining of very fœtid breath; obstructed nostrils and soreness upon pressure on the middle and side of his nose. The trouble commenced about four years ago,—(he is now about thirteen years of age), and was observed first during his convalescence from a severe attack of scarlatina, when it amounted only to an inconvenient running from the nostrils, which at the time, was not considered worth much attention. It has been gradually becoming worse, notwithstanding the use of various remedies, during the last three years. He is now very much troubled with the fœtor of the breath, soreness, bleeding and stuffing of the nostrils. He says, that in the morning when he first awakes, it takes half an hour's diligent work to so clear the nostrils that they permit the passage of air in the smallest quantity, and that from three to four times in the day must this painful and disagreeable operation be repeated,—each time causing more or less loss of blood. As far as can be seen up the nostrils, the mucous membrane is very red and much thickened; but I cannot observe any ulcerated patches, which may often be seen, nor can I learn that there has been any discharge of bone. But the probability is, judging, particularly from the disgusting fœtor of the breath, that there is ulceration of the membrane, and it is to be feared, that there is, also, caries of the bone, somewhere, deep in the numerous tortuous passages and cells which communicate behind the nasal openings. There are not many other affections with which ozœna is likely to be confounded.

You will more frequently be in doubt in distinguishing between it and polypus nasi; but, in addition to the circumstances that the polypus may nearly always be seen, when large enough to prove inconvenient, the obstruction from a polypus is permanent and not removable by the most strenuous efforts of the patient. There is, also, ordinarily very trifling, if any, fœtor in the case of polypus; while this symptom is early, persistent and prominent in ozœna.

I regard this case as one of the inflammatory sequelæ of scarlatina, and, at present, a local affection, unaccompanied by any important constitutional taint; so that I am now treating it with a very simple local remedy, which I have so frequently seen followed by successful results, that I very generally prescribe it as an item of treatment in most cases of this kind:

℞ Iodine, - - - - - grs.x.
Chloroform, - - - - ℥j.

Mix and dissolve.

This prescription is used by applying the open vial containing it, to one nostril, while the other is closed with the finger, so as to prevent the air from entering. As much of the vapor is inhaled, in this way, as can be taken with the inspired air, until the patient is conscious of a slight effect from the chloroform. He should now desist for ten minutes, and then inhale through the opposite nostril in the same way, and to the same extent. This should be repeated two or three times a day, persistently, for a length of time. The secretion of the schneiderian membrane is modified almost immediately upon the beginning of this inhalation. Although the boy has used this preparation but three days, as you hear him say, he is already much improved. This morning he had no more trouble in freeing his nostrils than could be accomplished by one or two ordinary efforts at blowing; no hard or inspissated mucus rendered the use of the finger and tweezers necessary. This is not more than the usual good effect I find from the use of this vapor. If the inflammation has not extended deep enough to destroy the integrity and function of the periosteum, the salutary effect wrought upon the membrane is sufficient to inaugurate the healing process in very many cases; while, should caries have already begun, it favorably influences the disease, so that as soon as that process is over, the further destruction is arrested. In specific conditions of the inflammation, specific constitutional remedies must accompany the use of the local. After the inhalation has been persevered in for several weeks, it often happens that its effects are less perceptibly beneficial, and the case comes to a stand-still, or retrogrades somewhat. When this happens, we should increase the proportion of iodine to the amount of chloroform,—say fifteen grains to the ounce,—and it may be thus gradually increased to ℈j., or ℈ij. to ℥j. Other remedies may be as beneficial, probably, as this one, when properly applied to the mucous membrane of the nose; but I think no other mode of using them, than by inhalations, will answer so well. The cavity of the nose is so complex, that injections but imperfectly reach many of its remote parts, if at all, as the stream cannot be made to follow all the tortuosities, or penetrate many of the cells connected with it. The volatile nature of iodine and chloroform enables us to impregnate, with their vapor, the air penetrating the nostril, and this vapor is carried wherever the air finds its way. Should there be any objection to the vapor passing the glottis, it can be snuffed with short, instead of deep inspirations; but the inflammation often affects, contemporaneously, if not continuously, the mucous membrane of the fauces, pharynx, larynx, and bronchi, and is just as beneficially influenced, by the remedy in those parts, as in the nose. The inhalation of remedial agents in the form of powders, such as cephalic snuffs, etc., although calculated to do good, is less efficacious than when vaporized.

